# Attitudes towards limited drug prescription rights: A survey of South African chiropractors

**DOI:** 10.4102/hsag.v27i0.1731

**Published:** 2022-02-11

**Authors:** Thriya Huluman, Christopher Yelverton, Cynthia Peterson

**Affiliations:** 1 Department of Chiropractic, Faculty of Health Sciences, University of Johannesburg, Johannesburg, South Africa

**Keywords:** attitudes, chiropractic, drug prescription, professional perspective, professional scope of practice

## Abstract

**Background:**

Several surveys that have been published show opinions regarding a change in the scope of chiropractic practice to include prescription rights. Currently, research into the attitudes of South African chiropractors towards having this right is non-existent.

**Aim:**

To ascertain the attitudes of South African chiropractors towards the inclusion of drug prescription rights in their scope of practice.

**Setting:**

The study was conducted on chiropractors registered with the Allied Health Professions Council of South Africa and members of the Chiropractic Association of South Africa.

**Method:**

A self-administrative online questionnaire was developed, and sent via email to all registered chiropractors in South Africa in February 2020. Descriptive statistics were used to analyse the data.

**Results:**

The response rate for this study was 15.9% (*n* = 138). 84% (*n* =105) were in favour of limited prescription rights for over-the-counter medication. However, 79.6% (*n* = 98) did not agree to full prescriptions rights for non-musculoskeletal drugs. A total of 33.6% (*n* = 42) rarely recommended OTC and prescription-based analgesics, muscle relaxants and non-steroidal anti-inflammatory drugs (NSAIDs) to their acute patients and 37.9% (*n* = 52) rarely recommended these drugs to chronic patients. 68.8% (*n* = 86) were confident in their knowledge of musculoskeletal drugs and 91.2% (*n* = 112) agreed on further education and training in pharmacology for those practitioners seeking limited medication prescription rights.

**Conclusion:**

The majority of South African chiropractor respondents indicated an interest in expanding their scope of practice to include limited prescription rights.

**Contribution:**

These findings could indicate a shift in the attitudes of chiropractors towards drug prescription rights within the profession.

## Introduction

The use of over-the-counter (OTC) and prescription drugs by chiropractors is a controversial issue worldwide (Emary & Stuber 2014). The World Federation of Chiropractic (WFC) (2013) has maintained the following policy statement on the use of prescription drugs:

[*F*]or reasons of chiropractic principle, patient welfare and interdisciplinary co-operation the practice of chiropractic does not include the use of prescription drugs, and chiropractic patients who may benefit from prescription drugs should be referred, where appropriate, to a medical doctor or other suitably qualified health care practitioners. (n.p.)

Emary and Stuber asserted that, in the approximately 120-year history of chiropractic, the profession has been known as a drugless and non-surgical healing profession (Emary & Stuber 2014), which has resulted in philosophic debates regarding the direction of the profession.

Despite these assertions, in some jurisdictions in the world, chiropractors can gain licences to prescribe OTC and/or prescription-based medications such as non-steroidal anti-inflammatory drugs (NSAIDs), analgesics and muscle relaxants for common musculoskeletal conditions (Robert 2003). At present, there are only two geographical regions that have limited prescriptions rights, namely Switzerland and the State of New Mexico in the United States (Emary & Stuber 2014). Studies have been published on this subject in the United States of America (McDonald, Durkin & Pfefer 2004), Canada (Emary & Stuber 2015), Australia (Jamison 1999) and the United Kingdom (Innes, Leboeuf-Yde & Walker 2016). However, in South Africa, research has only explored the current scope of practice of chiropractic in the country, but not the attitudes of chiropractors toward obtaining prescription rights.

Yet such rights are a requirement for those chiropractors within the chiropractic profession who are referred to as primary spine care providers, and fulfil their role as primary contact practitioners (Emary & Stuber 2015). The WFC defined chiropractors as experts in spinal health care in the health care system, which suggests that the WFC views chiropractors as primary spine care providers (Erwin, Korpela & Jones 2013). In order to be a primary spine care provider, it requires specialised training in spinal conditions, maintaining an evidence-based practice and acquiring a clear understanding of when and whom to refer appropriate patients to. The primary spine care provider should have adequate knowledge on the capabilities of other spine care providers that provide complementary interventions (Erwin et al. 2013). Nevertheless, prescribing drugs remains a contentious issue and the incorporation of these rights into the scope of chiropractic practice has significant implications for the profession (Emary & Stuber 2015).

In Switzerland, the chiropractic profession gained limited prescription rights in 1995 and has a high frequency of interprofessional referrals for spinal disorders. This practice is valuable and considered necessary for Swiss chiropractors as they are integrated with the healthcare system as primary spine care providers and recognised as one of the five medical professions in Switzerland (Humphreys & Peterson 2016; Innes et al. 2016). In the State of New Mexico, chiropractors have recently gained prescription rights and they also have access to diagnostic imaging such as computed tomography (CT) and magnetic resonance imaging (MRI) (Innes et al. 2016). In contrast, chiropractors in Canada have access to various limited diagnostic imaging procedures but do not have prescription rights.

Some lessons can be learned from the international experiences in Switzerland and the State of New Mexico and applied to chiropractic practice worldwide. In sum, to be recognised as a primary spine care provider, the following criteria need to be met: practice should (1) be evidence-based, (2) be scientifically justifiable, (3) be clinically relevant and (4) incorporate an integrated and collaborative approach to healthcare (Innes et al. 2016).

Evidence-based practice has been identified as the integration of research evidence with clinical skill as well as patient-related values. This approach should be widely adopted and supported by health professions (Innes et al. 2016). In order to practise responsibly, it is recognised that evidence-based practice is needed, as it increases the safety of patients and makes patient care more efficient. Evidence-based practice has decreased the length of stay in hospitals, increased survival rates, improved the quality of care, enhanced the quality of data and reduced healthcare costs (Erwin et al. 2013).

Because chiropractors in South Africa do not currently have prescription rights, the purpose of this study was to ascertain their attitudes towards attaining such rights.

## Methodology

### Study design

This was a quantitative study utilising a self-administered, online English questionnaire, which the registered chiropractors accessed via email through a link.

### Study setting

This study was conducted by means of an online survey questionnaire which was sent to all chiropractors registered with the Allied Health Professionals Council (AHPCSA) in South Africa.

### Study population and selection

A minimum of 100 responses were required for the data to be statistically significant, which is a minimum of 11.5% (confidence level of 95% with margin of error of 8%) of the total population size according to the statistician assigned to this study. There were 867 registered chiropractors at the time of circulation of the survey. The information letter and link to the survey were circulated to all members. Of these, 138 completed the survey, making the response rate for this study to be 15.9%. All respondents complied with the inclusion criteria.

### Questionnaire development

The questionnaire used for this study was based on Emary and Stuber (2014), who were consulted to adapt the questionnaire for the South African context. The changes in the survey included structural changes by which the survey prioritised the demographics. The institutions were changed to institutions that offer chiropractic qualifications in South African, and a question regarding philosophical orientation of chiropractors as ‘mixers’ and ‘straight’ chiropractors, was removed because of it not being relevant. In section 4, questions 10 and 11 were added with a list of different muscle relaxants and non-steroidal anti-inflammatories, to determine the level of usage by practitioners. The questionnaire was divided into four sections. Section 1 focused on demographic questions, including (1) age, (2) gender, (3) institution of graduation and (4) number of years in chiropractic practice or employment. Section 2 focused on questions related to the attitudes of South African chiropractors toward prescription-based drugs rights, the perceptions of chiropractors towards the prescription of either OTC medication or a limited number of prescription-based analgesics, non-steroidal anti-inflammatories or of any or all medications. The responses were recorded on a 5-point Likert scale ranging from ‘strongly disagree’ to ‘strongly agree’.

Section 3 of the questionnaire used a 5-point scale ranging from ‘never’ to ‘routinely’ and focused on the frequency of OTC drug recommendations currently taking place in chiropractic practice. The questions were centred on whether chiropractors recommended OTC medication to either an acute or chronic patient as well as how often the chiropractor refers a patient to a general practitioner for an anti-inflammatory drug or muscle relaxant.

Section 4 focused on questions related to chiropractors’ current knowledge of drug prescription practices (i.e. indications, contraindications, dosages and drug interactions). The responses were recorded on a 5-point Likert scale ranging from ‘very high’ to ‘very low’. The questions addressed chiropractors’ perceptions of their current knowledge on prescribing medication for musculoskeletal conditions versus non-musculoskeletal conditions, whether the chiropractor feels that there should be a post-graduate programme for pharmacology and drugs (muscle relaxants and NSAIDs) that they believe assist in pain relief and faster recovery.

There are various advantages to this methodology, some of which include cost-effectiveness and simplicity in distribution and analysis. The application of this method of survey design and distribution, provides for a user-friendly platform allowing for the exportation of data and ease in analysis. The platform used to deliver the survey was QuestionPro. Confidentiality and anonymity were maintained with the survey by not directly asking for the participants’ name or surname or any further confidential questions. A disadvantage was that various questions were misinterpreted and led to unanswered questions. As such, certain responses were disqualified, which impacted reliability.

### Pilot testing

A pilot survey was sent out to five chiropractors to pre-test the efficiency of the survey and to elicit feedback on the survey regarding face validity. These chiropractors were then excluded from the final survey. Pre-testing aided in identifying where corrections were required, ensuring that there were no grammatical errors and verifying that questions were not misunderstood or misinterpreted. The feedback allowed the researcher to make the necessary changes to ensure validity.

### Data analysis

The data was entered into the SPSS version 21 (IBM Corp, Armonk, NY), which was used to analyse the coded data. Descriptive analyses were used to explore the attitudes of chiropractors toward incorporating prescription rights into their scope of practice. The data was analysed by an independent entity, the statistician at the University of Johannesburg, STATKON division. Inferential statistics (Chi square) to determine the relationships between data were performed, but no significant differences were noted.

### Ethical considerations

This study was approved by the University of Johannesburg (UJ), Faculty of Health Sciences, Research Ethics Committee (REC -133-2019). The participants were provided with the study information in an invitation email and the survey cover sheet. The study information described the purpose of the survey and provided an estimate of the time required to complete the survey. Participation was voluntary and all data were anonymous, as no personal information was obtainable from the responses. The right to withdraw from the study at any time without consequence was stipulated. The participants provided anonymous consent by agreeing to a statement outlining the study information before being able to proceed to the survey.

## Results

The survey obtained 15.9% (*n* = 138) responses from qualified chiropractors in South Africa. After data cleaning, 14.4% (*n* = 125) of the responses were found to be valid and thus used for data analysis. Table 1 provides a summary of the demographic information of the study respondents including age, gender, university of graduation and number of years in practice. The survey statistics in Table 1 show the institutions of graduation, gender distribution, mean ages (standard deviation [SD]) of respondents and mean number of years in practice (SD).

**TABLE 1:** Demographic data of study respondents.

Variable	Total number of valid responses (*n*)	Results	
		SD	%
Mean (SD) years in practice	124	11	10.47
Mean age (SD) years	23	38.1	11.16
**Gender**	125	-	-
Male	57	45.6	-
Female	68	54.4	-
**University of graduation**	125	-	-
Technikon Witwatersrand	16	12.8	-
University of Johannesburg	52	41.6	-
Durban University of Technology	7	5.6	-
Natal Technikon	34	27.2	-
Other	7	5.6	-

SD, standard deviation.

The attitudes of South African chiropractors to drug prescription rights are summarised in Table 2. The majority of the respondents favoured the expansion of their scope of practice and allowing for the prescription of OTC medication for common musculoskeletal conditions. Similarly, the majority of respondents favoured the expansion of their scope of practice to allow for the prescription of a limited number of analgesics, anti-inflammatories and/or muscle relaxants. However, the majority of the participants were not in favour of having full prescription rights on any and all medication including controlled substances (e.g. antibiotics, anti-hypertensives, anti-depressants, corticosteroids etc.).

**TABLE 2:** Attitudes of South African chiropractors to drug prescription rights.

Attitudes	Agree		Disagree	
	*n*	%	*n*	%
Attitudes of chiropractors to prescribing OTC medication for musculoskeletal conditions	105	84	20	10.4
Attitudes of chiropractors to prescribing prescription-based analgesics, anti-inflammatories and/or muscle relaxants	94	75.2	31	19.2
Attitudes of chiropractors to prescribing all and any medication (e.g. antibiotics, anti-hypertensives, anti-depressants, corticosteroids etc.)	27	13	98	79.6

OTC, Over the counter.

**FIGURE 1:**
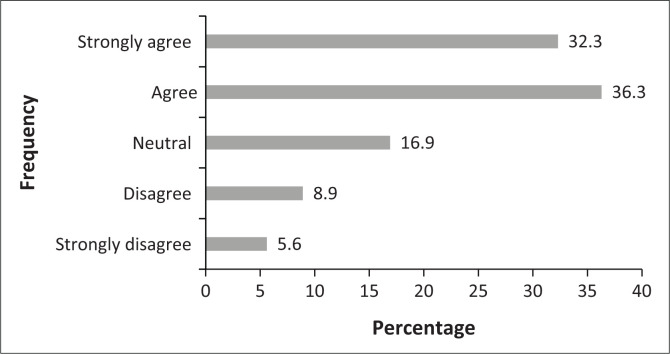
The beliefs of chiropractors towards over the counter drugs assisting in speeding up recovery of patients.

**FIGURE 2:**
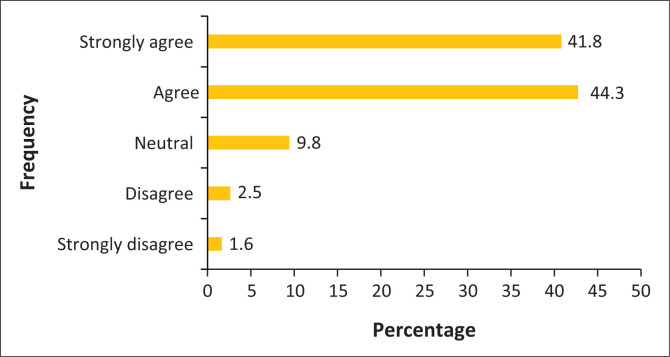
The attitudes of chiropractors towards over-the-counter drugs assisting in relieving pain of patients.

Most of the chiropractors who participated in the study believed that OTC drugs such as NSAIDs and muscle relaxants assist in speeding up the recovery of patients and a similar number of participants believed that OTC drugs, NSAIDs and muscle relaxants assist in relieving the pain of patients as shown in Figure 1 and Figure 2. Finally, the majority of respondents agreed on the importance of counselling patients on either the over-use of or the over-reliance on certain medications for musculoskeletal conditions.

**FIGURE 3:**
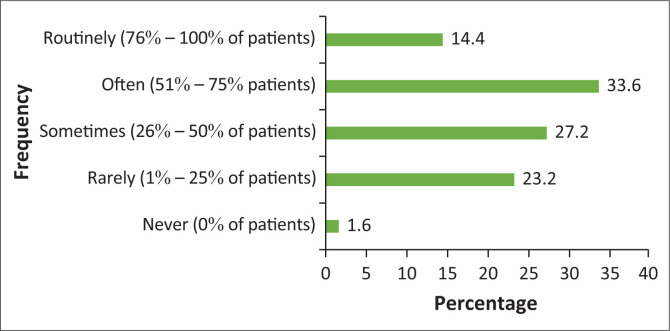
The frequency of over-the-counter drug recommendation to acute patients in chiropractic practice.

**FIGURE 4:**
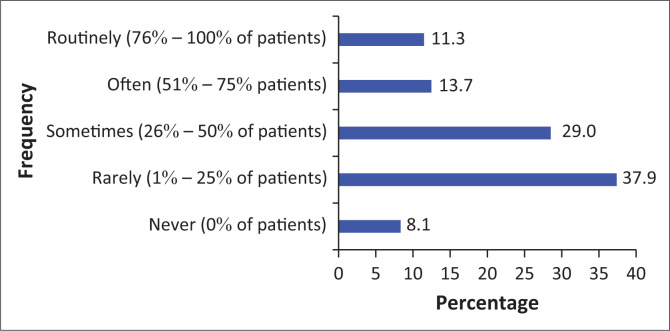
The frequency of over-the-counter recommendation to chronic patients in chiropractic practice.

**FIGURE 5:**
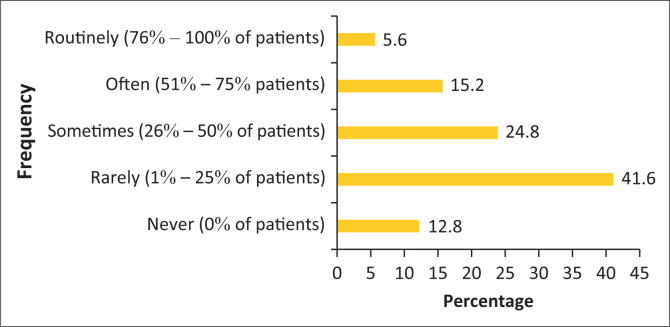
The frequency of referrals by chiropractors to general practitioners to receive an anti-inflammatory or muscle relaxant.

Section 3 of the survey focused on the frequency with which chiropractors recommend OTC medication to either acute or chronic patients in clinical practice. The responses revealed that 33.6% of the respondents often, which equates to 51% – 75% of the time, recommend OTC drugs to acute patients and 37.9% of the participants rarely, which equates to 1% – 25% of the time, recommend OTC drugs to chronic patients as seen in Figure 3 and 4. Section 3 of the survey also included a question on the frequency of referrals by South African chiropractors to general practitioners to receive an anti-inflammatory or muscle relaxant medication. This question showed that less than half of chiropractors (41.6%, or 1% – 25% of the time), rarely refer their patients to a general practitioner for these particular medications as seen in Figure 5.

Section 4 of the survey explored the self-reported current extent of knowledge of South African chiropractors on drug prescription. Table 3 illustrates that over two-thirds of South African chiropractors who responded to this survey were confident in their knowledge regarding prescribing musculoskeletal drugs, for example, analgesics, NSAIDs and muscle relaxants. Table 3 also illustrates that the vast majority of survey participants reported having less knowledge on prescribing drugs for non-musculoskeletal conditions, for example, antibiotics, anti-hypertensive drugs, anti-depressants, and so on.

**TABLE 3:** Current perceived knowledge of South African chiropractors on drug prescription.

Current knowledge	High		Low	
	*n*	%	*n*	%
Musculoskeletal drug prescription	86	68.8	39	31.2
Non-musculoskeletal drug prescription	22	17.6	103	82.4

The survey reflects whether South African chiropractors responding to this survey deemed it necessary to obtain a postgraduate certificate in pharmacology or drug administration in order to prescribe medication. In that regard, the vast majority (91.2%) of study participants believed that a postgraduate certificate programme in pharmacology or drug administration would be necessary for those in the profession who wish to prescribe drugs.

Table 4 illustrates the perceptions of participating South African chiropractors towards certain muscle relaxants and NSAIDs in relieving the pain of their patients. The vast majority favoured muscle relaxants methocarbamol acetaminophen (Robaxin), followed by orphenadrine citrate (Norflex), and NSAIDS Diclofenac and Ibuprofen for pain relief. In terms of perceptions towards muscle relaxants and NSAIDs speeding up the recovery of patients, muscle relaxant Baclofen was reported to be the least effective of the three options, with Diclofenac and Ibuprofen being perceived as superior.

**TABLE 4:** South African chiropractors’ perceptions on certain muscle relaxants and anti-inflammatories relieving pain and speeding up recovery of patients.

South African chiropractors perception response to	Total responses	No		Yes	
		*n*	%	*n*	%
**Certain muscle relaxants relieving pain of their patients**					
Baclofen, for example, Lioresal	112	44	39.3	68	60.7
Orphenadrine citrate, for example, Norflex	122	15	12.3	107	87.7
Methocarbamol acetaminophen, for example, Robaxin	118	20	16.9	98	83.1
**Certain muscle relaxants speeding up the recovery of their patients**					
Baclofen, for example, Lioresal	109	74	67.9	35	32.1
Orphenadrine citrate, for example, Norflex	115	62	53.9	53	46.1
Methocarbamol acetaminophen, for example, Robaxin	110	55	50	55	50
**Effectiveness of certain NSAIDs for reducing the pain of their patients**					
Acetylsalicylic acid, for example, Aspirin	117	35	29.9	82	70.1
Diclofenac, for example, Voltaren and/or Cataflam	121	5	4.1	116	95.9
Ibuprofen, for example, Nurofen	123	7	5.7	116	94.3
Naproxen sodium, for example, Alev	111	31	27.9	80	72.1
**Certain NSAIDs speeding up the recovery of their patients**					
Acetylsalicylic acid, for example, Aspirin	111	85	76.6	26	23.4
Diclofenac, for example, Voltaren and/or Cataflam	119	33	27.7	86	72.3
Ibuprofen, for example, Nurofen	117	47	40.2	70	59.8
Naproxen sodium, for example, Aleve	108	73	67.6	35	32.4

NSAIDS, non-steroidal anti-inflammatory drugs.

## Discussion

The primary findings of this study were that the majority of South African chiropractors who responded to this survey were in favour of expanding the scope of practice to include limited prescription rights. Although the response rate to this survey was above the minimum requirement for statistical significance, it was still lower than similar studies conducted in other countries. In a similar study conducted by Emary and Stuber in Ontario, the response rate was 22.9% (Emary & Stuber 2015). In Switzerland, where drug prescription rights for chiropractors are granted, the response rate was 77% (Wangler, Zaugg & Faigaux 2010). In the state of New Mexico, USA, another jurisdiction that has granted rights to prescribe drugs, the response rate was 10% (Lehman et al. 2011).

These findings are consistent with several previous international studies regarding the chiropractors’ rights to prescribe specific medications for their patients (Emary & Stuber 2015; Emary et al. 2016; Wangler et al. 2010). In the study conducted in Ontario, Canada, two-thirds of chiropractors agreed with having the right to prescribe OTC and prescription-based analgesics, NSAIDs and muscle relaxants (Emary & Stuber 2015). In the study conducted in Switzerland, 72% of Bernese (a canton of Switzerland) chiropractors were in agreement that it would be an advantage for the chiropractic profession to be able to prescribe medication, and 95% of the respondents supported the prescription of NSAIDs, muscle relaxants and additional analgesics (Wangler et al. 2010). As indicated by Lehman et al., in the state of New Mexico, USA, patients’ perspectives of whether qualified chiropractors should have prescriptive authority found that 85% of the patient respondents preferred that their chiropractor was qualified to prescribe medication as well as provide hands-on treatment in order to control pain (Lehman et al. 2011).

Emary et al. stated in a commentary on the implications of medication prescription rights for the chiropractic profession that the arguments against prescribing rights mainly include the divisiveness within the profession around this topic (Emary et al. 2016). Although there is a split in the opinions about prescription rights in chiropractic practice, there is evidence that this split relates to differing philosophical ideologies within the profession and that those who are against this right are in the minority (Emary et al. 2016).

Irrespective of these philosophical differences, there is sufficient evidence from the increasing number of published surveys that suggests that there appears to be a change in chiropractors’ attitudes toward prescription rights, with more chiropractors’ favouring an expansion in their scope of practice to include specific prescription rights. Surveys conducted in Australia (Jamison 1999), New Mexico (McDonald et al. 2004), and the United Kingdom (Innes et al. 2016) have found that 54% – 78% of respondents showed an interest in having prescription rights for selective medications, including NSAIDs, analgesics and muscle relaxants (Lehman et al. 2011). However, as stated by Gliedt et al., in North America, the student respondents were not in favour of expanding the scope of practice to include prescription rights (Gliedt et al. 2015). Similar student surveys have not been carried out outside of the US and those results would likely be different from the US student responses because the educational programmes that promote chiropractic treatments without the use of medications are mainly located in the US. Follow-up studies in other countries would be very informative.

The over-use and over-reliance on specific pain medications such as opioids are increasingly becoming an important issue to address as there is an over-prescription of these types of drugs in the US and many other countries (Gliedt et al. 2015). The Gleidt et al. study showed that 83.3% of the chiropractic student respondents agreed that chiropractors should counsel patients on either the over-use or the over-reliance on medication for musculoskeletal conditions. The ability of chiropractors to counsel patients on this matter is important and practitioners should be interested in it regardless of their philosophical orientation. As stated by Wangler et al. in Switzerland, chiropractors prescribe medication less often than they are requested to by their patients (Wangler et al. 2010).

Emary et al. stated in their commentary on the implications of medication prescription rights in the chiropractic profession that one of the arguments in favour of having limited prescriptive authority was a positive influence on public health (Emary et al. 2016). They further indicated that NSAIDs and analgesics are generally widely used and potentially misused by the public, yet the potential side effects of such medication are not known by the users (Emary et al. 2016). Therefore, if granted prescriptive authority, chiropractors would be in the position to advise patients on the proper use of these types of medication. This perception is consistent with the current best-practice guidelines, with which chiropractors resonate (Emary et al. 2016).

### Frequency of drug recommendations

This study showed that less than half of the respondents often (51% – 75%) recommend OTC drugs to acute patients and less than half of the respondents rarely (1% – 25%) recommend OTC drugs to chronic patients. As indicated by Wangler et al. in Switzerland, 72% of their respondents agreed that medications are necessary in extremely acute cases where absolutely no range of motion can be achieved (Wangler et al. 2010).

This study also showed that less than half of the respondents reported that they rarely (1% – 25%) refer their patients to a general practitioner to receive anti-inflammatory or muscle relaxant medications. Having limited prescription rights could benefit the profession’s efficiency and reduce the patient’s time and money spent on healthcare services. As indicated by Emary et al., having limited prescription authority could facilitate the chiropractic profession having better integration into the healthcare system (Emary et al. 2016). Members of a profession who are both skilled in manual therapy and have limited prescriptive authority could play a significant role in the evidence-based care of spinal and musculoskeletal conditions. Such a change in scope of practice has the potential to benefit not only patients but also the chiropractic profession by improving its identity and increasing its responsibility. In Switzerland, where the profession has limited prescription rights as a privilege within its scope of practice, chiropractors are fully integrated into the healthcare system as one of the five regulated medical professions (Gliedt et al. 2015; Humphreys & Peterson 2016).

### South African chiropractors’ current knowledge of pharmacology

In this study, most of the respondents were confident in their knowledge of musculoskeletal drugs but were not very confident in their knowledge of non-musculoskeletal drugs. Therefore, almost all of the respondents felt it necessary to obtain a postgraduate certificate in pharmacology or drug administration in order to be allowed to prescribe medication. These results corresponded with those of the study conducted in Ontario, Canada, where two-thirds of the respondents rated their knowledge on prescribing drugs for musculoskeletal conditions as ‘high’ to ‘very high’ (Emary & Stuber 2015) and nearly equal numbers responded to having ‘low’ to ‘very low’ knowledge on prescribing drugs for non-musculoskeletal conditions (Emary & Stuber 2015). Currently, chiropractors in South Africa complete pharmacology in their third year of study at the UJ as a year module and in their fourth year at Durban University of Technology (DUT) as a semester module. It is also relevant to point out that podiatrists graduating from the UJ complete the same course in pharmacology as the chiropractic students, but are given prescription rights whereas the regulations for chiropractors currently prohibit similar prescription privileges.

In New Mexico, USA, where chiropractors currently have limited prescriptive authority, chiropractors must complete a 2-year postgraduate Master of Science degree in Advanced Clinical Practice before they can obtain the licence to prescribe a limited formulary in that state. Further training in pharmacology is offered in this postgraduate programme and it serves as a model for the chiropractic profession for other jurisdictions trying to obtain these rights (Emary & Stuber 2015).

Indeed, education and training are a concern when considering any expansion in the scope of practice in the chiropractic profession. As stated by Lehman et al., the institution that was considering to expand the training and education for chiropractors in New Mexico, USA was the National University of Health Sciences in Chicago, which included a 2-year Master’s degree in Advanced Practice (Lehman et al. 2011). Lehman et al. reported that, according to the New Mexico Statutes Annotated (NMSA), chiropractic physicians can seek requisite training for credentials that permit limited prescriptive authority (Lehman et al. 2011) in that state. Those with active registration will be allowed prescription authority on only the agreed formulary approved by the New Mexico Board of Chiropractic Examiners, the New Mexico Board of Pharmacy and the New Mexico Medical Board (Wangler et al. 2010).

In Switzerland, 91% of the respondents stated that chiropractors should attend mandatory classes before they prescribe NSAIDs, muscle relaxants and analgesics. As stated by Wangler et al., the comments by the respondents were focused on continuing education in pharmacology and emphasised understanding dangers and possible interactions or side effects of medication. Because this field is often changing, continuing education is essential to remain updated with the latest knowledge (Wangler et al. 2010). Switzerland is unique in that it has a 3-year, full-time, post-graduate residency programme, which chiropractors must attend in order to be allowed to practise independently (Humphreys & Peterson 2016). One of the modules in this programme is on the pharmacological products that Swiss chiropractors are allowed to prescribe. Furthermore, graduates of the 6-year Master’s in Chiropractic Medicine programme (MChiroMed) at the University of Zurich attend classes that comprise the first 4 years of medicine along with their medical student colleagues, including all pharmacology courses (Humphreys & Peterson 2016).

Although there was a high interest in prescribing drugs and completing a postgraduate certificate in pharmacology by South African chiropractors, 79.6% (*n* = 98) of the respondents in this study were not in favour of having full prescription rights for any and all medication including controlled substances for non-musculoskeletal conditions (e.g. antibiotics, anti-hypertensives, anti-depressants, corticosteroids, narcotics etc.). These findings corresponded with the results of the study conducted in Ontario, Canada, where more than three-quarters of the respondents did not want to expand the scope of practice to allow rights to prescribe all and any medication (Emary & Stuber 2015). Findings from previous surveys conducted in Australia and the United States were similar (Jamison 1999; McDonald et al. 2004). This restriction influences the breadth and depth of the education leading to the postgraduate certificate. Indeed, many chiropractors may not want to complete more than the minimum requirements as they would not want to prescribe an extended formulary of drugs for non-musculoskeletal conditions.

The respondents of this study were also asked about their perceptions of whether or not certain drugs either relieved pain or sped up the recovery of their patients. Almost all of the respondents agreed that the muscle relaxant Robaxin was more helpful in relieving the pain of their patients compared to the drugs Norflex and Lioresal. However, this data is only based upon personal opinion and not upon any previously published research. The general consensus of this study is that the respondents did not agree on whether or not muscle relaxants speed up the recovery of their patients. The respondents’ perception regarding NSAIDs in terms of relieving the pain of their patients revealed that the majority agreed that Voltaren (diclofenac sodium) and/or Cataflam (diclofenac potassium) most effectively relieved the pain of their patients, closely followed by Nurofen (ibuprofen). Furthermore, the respondents felt that these two drugs were also more effective in speeding up the recovery of their patients compared to Aleve (naproxen) and Aspirin (acetylsalicylic acid).

This study showed that chiropractors may support obtaining prescribing rights in South Africa. Evidence from the literature and results from the current study suggest that among chiropractors who hold favourable views toward drug prescription, prescription privileges limited to a musculoskeletal scope of practice would be preferred (Emary & Stuber 2015). A large majority of respondents in the current study also agreed that, with limited prescriptive authority chiropractors could advise patients against overusing analgesics and anti-inflammatory medications. Evidence that supports this belief can be found in Switzerland, where chiropractors tend to prescribe medications significantly less often than requested by their patients (Humphreys & Peterson 2016). Further surveys and/or qualitative studies of chiropractors’ opinions toward gaining prescription privileges would be necessary. If similar findings are confirmed elsewhere, there would be cause for a national campaign to reform the chiropractic scope of practice across the country.

### Limitations

The response rate of 14.4% met the statistical significance (10%) for this study according to the statistician at the UJ but was lower in comparison to the international standards and similar studies conducted. Reminder notifications for the completion of the survey were not sent out as done in other countries, because of the protocols set out in the distribution of the survey from the AHPCSA at that stage. Thus, the results of this study may be because of a sampling bias if those chiropractors who were more interested in having limited prescription rights were more inclined to respond.

Furthermore, the data obtained on whether or not specific medications do result in more pain reduction or speed up recovery compared to other medications are only the perception of the participating chiropractors.

## Conclusion

This study revealed that the majority of the South African chiropractors responding to this survey were in favour of having limited medication prescription rights within the chiropractic scope of practice. They were generally confident in their knowledge of medications regarding musculoskeletal conditions but also thought that additional post-graduate education should be required.

Further studies are needed to explore dialogue among professional bodies and chiropractic regulatory authorities to consider the expansion of the scope of practice of the profession to include limited prescription rights with the requisite training and legislation.
